# Real-time imaging of bHLH transcription factors reveals their dynamic control in the multipotency and fate choice of neural stem cells

**DOI:** 10.3389/fncel.2015.00288

**Published:** 2015-08-04

**Authors:** Itaru Imayoshi, Fumiyoshi Ishidate, Ryoichiro Kageyama

**Affiliations:** ^1^The Hakubi Center, Kyoto UniversityKyoto, Japan; ^2^Laboratory of Growth Regulation, Institute for Virus Research, Kyoto UniversityKyoto, Japan; ^3^World Premier International Research Initiative–Institute for Integrated Cell-Material Sciences, Kyoto UniversityKyoto, Japan; ^4^Precursory Research for Embryonic Science and Technology, Japan Science and Technology AgencySaitama, Japan; ^5^Core Research for Evolutional Science and Technology, Japan Science and Technology AgencySaitama, Japan

**Keywords:** bHLH, neural stem cells, oscillation, fluorescent protein, imaging

## Abstract

The basic-helix-loop-helix (bHLH) transcription factors Ascl1/Mash1, Hes1, and Olig2 regulate the fate choice of neurons, astrocytes, and oligodendrocytes, respectively; however, these factors are coexpressed in self-renewing multipotent neural stem cells (NSCs) even before cell fate determination. This fact raises the possibility that these fate determination factors are differentially expressed between self-renewing and differentiating NSCs with unique expression dynamics. Real-time imaging analysis utilizing fluorescent proteins is a powerful strategy for monitoring expression dynamics. Fusion with fluorescent reporters makes it possible to analyze the dynamic behavior of specific proteins in living cells. However, it is technically challenging to conduct long-term imaging of proteins, particularly those with low expression levels, because a high-sensitivity and low-noise imaging system is required, and very often bleaching of fluorescent proteins and cell toxicity by prolonged laser exposure are problematic. Furthermore, to analyze the functional roles of the dynamic expression of cellular proteins, it is essential to image reporter fusion proteins that are expressed at comparable levels to their endogenous expression. In this review, we introduce our recent reports about the dynamic control of bHLH transcription factors in multipotency and fate choice of NSCs, focusing on real-time imaging of fluorescent reporters fused with bHLH transcription factors. Our imaging results indicate that bHLH transcription factors are expressed in an oscillatory manner by NSCs, and that one of them becomes dominant during fate choice. We propose that the multipotent state of NSCs correlates with the oscillatory expression of several bHLH transcription factors, whereas the differentiated state correlates with the sustained expression of a single bHLH transcription factor.

## Introduction

Neural stem cells (NSCs) are multipotent and self-renewable cells that can give rise to neurons, astrocytes, and oligodendrocytes (Fishell and Kriegstein, 2003; Götz and Huttner, 2005; Kriegstein and Alvarez-Buylla, 2009). During brain development, basic helix-loop-helix (bHLH) transcription factors play pivotal roles in the self-renewal of NSCs and fate determination of neurons, astrocytes, and oligodendrocytes (Bertrand et al., 2002; Ross et al., 2003; Meijer et al., 2012; Wilkinson et al., 2013; Imayoshi and Kageyama, 2014a). bHLH transcription factors, such as Hes1 and Hes5, regulate the self-renewal of NSCs as downstream effectors of Notch signaling, whereas proneural bHLH transcription factors, such as Ascl1 and Neurog1/2, promote neuronal differentiation. Other bHLH transcription factors, e.g., Olig1 and Olig2, regulate oligodendrocyte differentiation. However, in addition to Hes1/Hes5, some bHLH fate determination factors, such as Ascl1 and Olig2, are also known to have roles in NSC maintenance or proliferation. In addition, Hes1 induces astrocyte formation at later stages. Currently, it is not understood completely how these various and sometimes opposing functions of each bHLH transcription factor in the self-renewal and fate-choice events of NSCs are achieved (Vasconcelos and Castro, 2014; Imayoshi and Kageyama, 2014a,b). We previously found that transcription of some bHLH genes is differentially controlled in NSCs and differentiating cells (Shimojo et al., 2008). Therefore, we decided to analyze the expression dynamics of bHLH transcription factors in more detail.

## Heterogeneous expression of bHLH transcription factors in cultured NSCs

NSCs can be cultured in an undifferentiated state *in vitro*. For instance, in the presence of epidermal growth factor and basic fibroblast growth factor, NSCs can be expanded from enzymatically dissociated brain tissues in a monolayer condition (NS cell culture) (Conti et al., 2003). NSCs can reportedly be established from brains at various stages, from embryos to adults. Furthermore, the purity of these cultures is very high; more than 99% of cells express NSC marker proteins, such as Nestin. Cultured NSCs also express bHLH transcription factors, including Hes1, Ascl1, and Olig2 (Imayoshi et al., 2013a).

**Figure 1:**
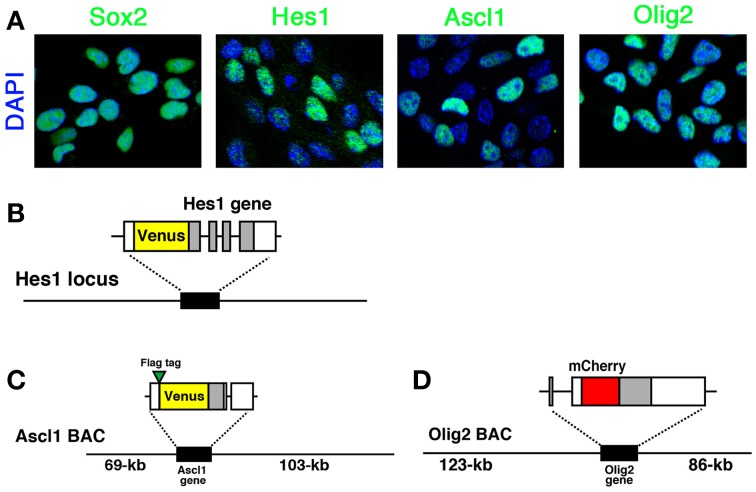
**Expression of bHLH factors in NS cells and fluorescent reporter-bHLH fusion constructs**. **(A)** Self-renewing NS cells were immunostained with anti-Sox2, anti-Hes1, anti-Ascl1, and anti-Olig2 antibodies. Hes1, Ascl1, and Olig2 expression levels were variable while another NSC-specific factor, Sox2, was expressed at a relatively constant level. **(B)** Venus-Hes1 fusion knock-in mouse strain. **(C)** Venus-Ascl1 fusion BAC Tg mouse strain. **(D)** mCherry-Olig2 fusion BAC Tg mouse strain. Coding regions of bHLH factors are shown in gray. The pictures and graphs of this figure are reprinted from Supplementary Figures S1C,E; S2A; S6A–D of Imayoshi et al. (2013b).

In actively-dividing and self-renewing NS cells, Hes1, Ascl1, and Olig2 bHLH transcription factors are expressed (Figure 1A). Compared with the homogeneous expression of Sox2, which is a member of the high-mobility-group transcription factor family, the expression of all three bHLH transcription factors is variable among cells, indicating their dynamic expression (Figure 1A) (Imayoshi et al., 2013b). In order to elucidate the heterogeneous expression of bHLH transcription factors, we adopted a real-time imaging strategy to monitor the expression dynamics of bHLH proteins in cultured NSCs.

## Fluorescent reporter-fusion knock-in or bacterial artificial chromosome (BAC) transgenic (Tg) strategy for monitoring protein expression dynamics of bHLH transcription factors

Fusion reporter constructs are used to monitor the expression dynamics of proteins of interest in living cells (Figures 1B–D) (Miyawaki, 2011; Imayoshi et al., 2013a). In addition to fluorescent reporter proteins, bioluminescent reporters, such as luciferase, are also effective. One advantage of the reporter-fusion strategy is that the half-life and cellular localization of proteins basically depend on the fused target protein, at least in the case of bHLH transcription factors. Most bHLH transcription factors have very short half-lives, such as ~20 min, and therefore reporter-bHLH fusion proteins are also very unstable.

In order to analyze precisely the fine expression dynamics of bHLH transcription factors, it is necessary that reporter-bHLH fusion proteins are expressed under the control of their original promoter/enhancer sequences. One of the reliable ways to mimic the original regulation of bHLH gene expression is the knock-in strategy (Imayoshi et al., 2013a,b). By homologous recombination in embryonic stem cells, a reporter coding sequence is inserted into the N− or C− terminus of a bHLH gene so that the reporter-bHLH fusion protein is expressed from the original bHLH gene locus. As far as we know, the reporter-bHLH fusion protein is functional and, in most cases, the allele carrying the knocked-in fusion reporter functions as normally as the wild-type allele. Another strategy to express reporter-bHLH fusion proteins optimally is the generation of Tg mice with a BAC. Using homologous recombination in *Escherichia coli*, we can edit and modify BACs (Lee et al., 2001). BACs can contain very long DNA sequences, such as ~200-kb. Therefore, by utilizing BAC clones with the promoter/enhancer sequences of a bHLH gene, it is possible to make transgene constructs designed to express reporter-bHLH fusion proteins under its original regulatory elements. From the Tg mouse founder lines carrying BAC transgene constructs, we can choose ones with a transgene copy number of only one by Southern blotting or real-time PCR analysis. These one-copy BAC Tg mice work as reporter strains for real-time imaging of the expression dynamics of bHLH transcription factors (Imayoshi et al., 2013b). One concsern regarding the reporter-fusion BAC Tg mouse strategy is that the number of functional bHLH gene alleles is increased from two to three. Ideally, reporter expression should be analyzed in a heterozygous-mutant background of the target gene by crossing with a mouse strain harboring a null allele. However, at least in the case of bHLH transcription factors, such as Hes1, Ascl1, or Olig2, the addition of the reporter-fusion transgenes dose not apparently affect the normal development of the nervous system (Imayoshi et al., 2013b).

As far as we know, the N-terminal fusion of a reporter proteins in both knock-in and BAC Tg mice shows better results than for a C-terminal fusion. The fluorescence or bioluminescence of a reporter protein is brighter when it is fused to the N-terminus than to the C-terminus of bHLH transcription factors. This is probably because translation starts from the N-terminus, so a reporter protein located at the N-terminus starts to work earlier during its expression.

For quantitative live-imaging of fluorescent reporters, we established NS cell cultures from each knock-in or BAC Tg mouse strain (Imayoshi et al., 2013b). In fluorescent protein-bHLH fusion reporter mice, Venus-Hes1 fusion knock-in, Venus-Ascl1 fusion BAC Tg, and mCherry-Olig2 fusion BAC Tg mouse strains (Figures 1B–D), the expression levels of fusion proteins and endogenous bHLH proteins were highly correlated to each other in cultured NSCs (Imayoshi et al., 2013b). Therefore, we conducted real-time imaging of fluorescent protein-bHLH fusion reporters in cultured NSCs with confocal fluorescence microscopy.

## Real-time imaging of fluorescent reporter-fusion bHLH proteins with confocal fluorescent microscopy

For real-time imaging of fluorescent proteins with low expression levels, it is important to prepare a highly sensitive and low-noise imaging system. As we mentioned earlier, the mRNA and protein of most bHLH transcription factors have very short half-lives (e.g., ~20 min): therefore, the reporter-bHLH fusion proteins are also very unstable (Imayoshi et al., 2013a,b). We can measure the decay time of reporter-bHLH fusion products by pharmacologically blocking the synthesis of new cellular proteins with cycloheximide. Indeed, the reporter activity of bHLH-fusion products are decayed with the same time course as the original bHLH proteins (data not shown). Furthermore, in cultured NSCs derived from knock-in or BAC Tg mice, reporter-bHLH fusion proteins are expressed from a limited number of gene alleles. Therefore, the fluorescent signals of reporter-bHLH fusion proteins in NSCs are very dim and sometimes invisible by simple observation through ocular lenses.

To acquire these very weak fluorescent signals, we utilized a confocal microscope equipped with a highly sensitive detector (Imayoshi et al., 2013b). In some cases, the fluorescent signal intensity of reporter-bHLH fusion proteins is much weaker than that of the autofluorescence of cultured NSCs. To overcome this problem, a spectral imaging technique is very effective. Spectral imaging and linear unmixing has become an important tool in confocal fluorescence microscopy to discriminate between fluorescent signals with overlapping spectral characteristics. For example, when cultured NSCs are illuminated with a 514-nm argon laser, the autofluorescent signal appears at approximately 560 nm. Real signals from Venus-Hes1 or Venus-Ascl1 fusion proteins are observed approximately 530 nm, and their intensity is weaker than the autofluorescence of NSCs. However, when the fluorescent signals are acquired with a spectral GaAsP array detector, specific Venus-bHLH signals can be separated from autofluorescence by applying linear unmixing algorithms.

In addition to the highly sensitive and spectral imaging techniques, imaging systems should be optimized for low-noise and reliable quantitative analysis. As the fluorescent signal intensity of reporter-bHLH fusion proteins is very weak, contamination with noise signals impairs the reliable quantitative analysis of the expression dynamics of bHLH transcription factors. To prevent bleaching the fluorescent reporters and avoid photodamage to NSCs by the excitation lasers, the sensitivity of the imaging system is again critically important. Bleaching of the reporters impairs the reliability of the imaging results. During long-term continuous imaging, laser power should be as small as possible, because NSCs are delicate and very sensitive to exogenous perturbations, including photodamage by exposure to a strong laser.

## Oscillatory expression of bHLH transcription factors in multipotent NSCs

**Figure 2:**
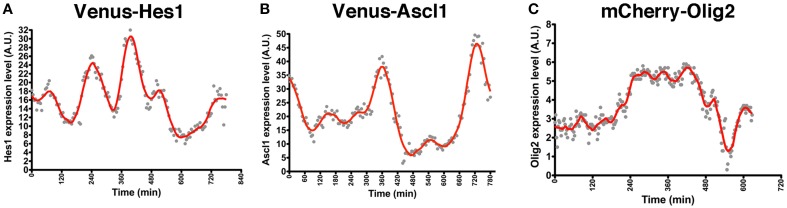
**Oscillatory expression of bHLH factors in NSCs**. NS cell cultures were prepared from Venus-Hes1 knock-in mice **(A)**, Venus-Ascl1 BAC Tg mice **(B)**, or mCherry-Olig2 BAC Tg mice **(C)**. **(A–C)** Examples of quantification of each fusion protein dynamics in self-renewing NSCs. The graphs of this figure are reprinted from Supplementary Figures S8C; S11G; S12D of Imayoshi et al. (2013b).

NS cell cultures established from Venus-Hes1 fusion knock-in, Venus-Ascl1 fusion BAC Tg, and mCherry-Olig2 fusion BAC Tg mouse strains were used for real-time imaging with confocal fluorescence microscopy. This analysis revealed that the fluorescent fusion reporter proteins with Hes1, Ascl1, or Olig2 exhibited dynamic expression changes in NS cells (Supplementary Movies 1–3), as observed in the bioluminescence imaging experiments with luciferase-bHLH fusion reporters (Imayoshi et al., 2013b). By quantitative analysis of single cells, the levels of Hes1 and Ascl1 proteins seem to oscillate with a 2–3-h period (Figures 2A,B), while Olig2 oscillates with a longer period, such as 5–8 h (Figure 2C) (Imayoshi et al., 2013b). Although fluorescent reporter-bHLH proteins showed dynamic expression changes, oscillatory expression was observed more clearly in the bioluminescence imaging experiments with luciferase-bHLH fusion reporters. Especially, the peak amplitude during signal changes was larger in the case of luciferase-bHLH fusion reporters (Imayoshi et al., 2013b), which is partly because the protein maturation time of luciferase is more rapid than that of fluorescent proteins, such as Venus and mCherry. Our observations revealed that, in actively dividing and multipotent NSCs, bHLH transcription factors are expressed in an oscillatory manner.

The oscillatory expression of bHLH transcription factors raised the possibility that NSCs may change their fate preferences over time. Indeed, when cultured NSCs are sorted by their expression levels of each bHLH transcription factor, their differentiation preferences are correlated with bHLH expression levels (Imayoshi et al., 2013b). For instance, Hes1-high NSCs preferentially differentiate into astrocytes, whereas Ascl1-high and Olig2-high NSCs preferentially differentiate into neurons and oligodendrocytes, respectively. These results suggest that different expression levels of bHLH transcription factors bias the fate choices of NSCs. However, such transient high expression of bHLH transcription factors may not be sufficient for cell fate determination, because these NSCs are still multipotent. Thus, we analyzed how the expression of these bHLH transcription factors changes during cell fate choice.

## Expression dynamics of bHLH transcription factors in multipotency and fate choice

**Figure 3:**
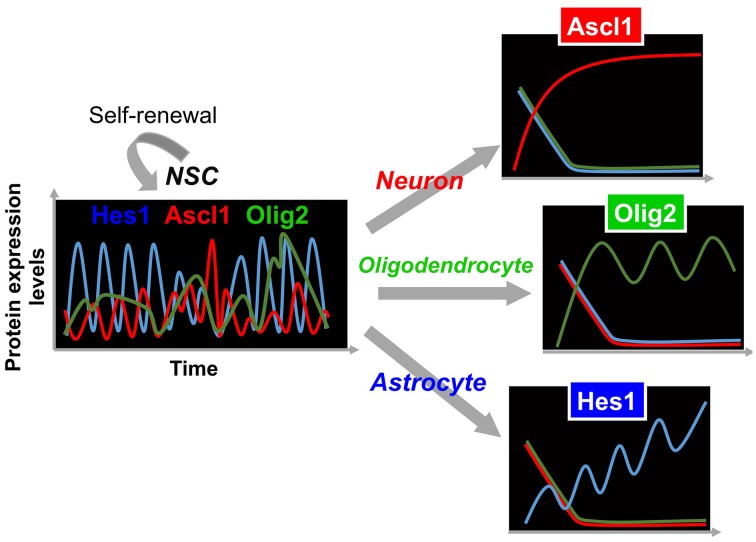
**Expression dynamics of bHLH factors in multipotency and cell fate choice**. In multipotent NSCs, the levels of Hes1 and Ascl1 oscillate with periods of 2–3 h, while that of Olig2 oscillates with a period of 5–8 h. By contrast, during cell fate choice, one of the bHLH factors is expressed in a sustained manner, while the others are repressed.

In contrast to oscillatory expression of multiple bHLH transcription factors in self-renewing NSCs, one of the bHLH transcription factors is expressed in a sustained manner during cell fate choice, while the others are repressed (Figure 3) (Imayoshi et al., 2013b). For instance, we found that the transient down-regulation of Hes1 expression and the concomitant up-regulation of Ascl1 before cell division bias NSCs toward a neuronal fate choice, and the sustained expression of Ascl1 after cell division irreversibly determines neuronal fate (Figure 3). During astrocyte and oligodendrocyte differentiation, the expression of Hes1 and Olig2 is upregulated, respectively, although they still oscillate. However, even during the trough phases, both Hes1 and Olig2 levels are higher than they are in NSCs, indicating that Hes1 and Olig2 expression continues in a sustained manner during astrocyte and oligodendrocyte differentiation (Figure 3). When Hes1 or Olig2 becomes dominant, the expression of the other two factors is downregulated. These results indicate that Hes1, Ascl1, and Olig2 are expressed in an oscillatory manner in multipotent NSCs, and that one of them becomes dominant during cell fate choice (Imayoshi et al., 2013b).

From these analyses, we propose the following model: the oscillatory expression of multiple bHLH transcription factors is correlated with the multipotent and self-renewable state of NSCs, whereas the sustained expression of a selected bHLH transcription factor regulates fate determination (Figure 3) (Imayoshi et al., 2013b; Imayoshi and Kageyama, 2014a,b). To validate this model, we applied a new optogenetic method (photo-activatable Gal4/UAS system) to manipulate artificially the expression patterns of bHLH transcription factors using blue light illumination, showing that oscillatory expression activates the proliferation of NSCs, whereas sustained expression induces cell fate determination. Detailed results are reviewed elsewhere (Imayoshi et al., 2013b; Imayoshi and Kageyama, 2014a,b).

## Conclusion

Previous reports have shown some evidence that the bHLH transcription factors Hes1, Ascl1, and Olig2 have multiple functions, such as NSC proliferation, self-renewal, and differentiation. Of course, protein modifications and/or partner co-factor variation may be involved in these different activities of bHLH transcription factors in a complex manner, but our studies revealed that oscillatory versus sustained expression dynamics also contribute to these different functions. More generally, our studies indicated that expression patterns, rather than simply the expression levels, of various transcription factors regulate whether stem cells proliferate or differentiate. In addition to Hes1, Ascl1, and Olig2, other bHLH transcription factors, such as Hes5 and Neurog2, are expressed in an oscillatory manner in NSCs or neural precursor cells (Shimojo et al., 2008; Imayoshi et al., 2013b). Dynamic expression is not necessarily confined to bHLH-type transcription factors, and many kinds of transcription factors are known to be involved in NSC regulation: therefore, it is expected that future imaging and manipulation studies will unveil the unique expression dynamics and their significance of various transcription factors in NSCs.

bHLH transcription factors regulate other kinds of somatic stem cells and pluripotent stem cells. For example, Ascl1 is essential for the differentiation of neuroendocrine cells from epithelial progenitors of the developing glandular stomach (Kokubu et al., 2008), and cyclical Hes1 expression in embryonic stem cells is important for their diverse differentiation abilities (Kobayashi et al., 2009). Therefore, the dynamic control of bHLH transcription factors, including their oscillatory expression, may regulate many regulatory processes of other kinds of stem cells.

Our studies suggest the importance and future possibilities of imaging techniques for the analysis of the expression dynamics of endogenous proteins at the single-cell level by coupling with various cellular events, such as cell cycle progression and cell differentiation (Miyawaki, 2011; Isomura and Kageyama, 2014). Under the current situation in developmental biology in which many biological processes start to be understood from the viewpoint of systems biology or single-cell biology (Levine et al., 2013; Sanchez and Golding, 2013; Zenobi, 2013), it is expected to become even more important to image subcellular localization and expression dynamics over time at a high resolution while keeping the original expression levels of target proteins.

### Conflict of interest statement

The authors declare that the research was conducted in the absence of any commercial or financial relationships that could be construed as a potential conflict of interest.

## Supplementary material

Supplementary Movie 1 **Venus-Hes1 expression in self-renewing NSCs**. Time in h and min is indicated. Related to Figure 2A.

Supplementary Movie 2 **Venus-Ascl1 expression in self-renewing NSCs**. Time in hr min, and sec is indicated. Related to Figure 2B.

Supplementary Movie 3 **mCherry-Olig2 expression in self-renewing NSCs**. Time in hr min, and sec is indicated. Related to Figure 2C.
